# Suicide prevention in primary care: General practitioners' views on service availability

**DOI:** 10.1186/1756-0500-3-246

**Published:** 2010-10-01

**Authors:** Pooja Saini, Kirsten Windfuhr, Anna Pearson, Damian Da Cruz, Caroline Miles, Lis Cordingley, David While, Nicola Swinson, Alyson Williams, Jenny Shaw, Louis Appleby, Navneet Kapur

**Affiliations:** 1 National Confidential Inquiry into Suicide and Homicide by People with Mental Illness, Centre for Suicide Prevention, Jean McFarlane Building, University of Manchester, M13 9PL, UK; 2 Centre for Criminology, University of Oxford, Manor Road Building, Manor Road, Oxford, OX1 3UQ, UK

## Abstract

**Background:**

Primary care may be a key setting for suicide prevention. However, comparatively little is known about the services available in primary care for suicide prevention. The aims of the current study were to describe services available in general practices for the management of suicidal patients and to examine GPs views on these services. We carried out a questionnaire and interview study in the North West of England. We collected data on GPs views of suicide prevention generally as well as local mental health service provision.

**Findings:**

During the study period (2003-2005) we used the National Confidential Inquiry Suicide database to identify 286 general practitioners (GPs) who had registered patients who had died by suicide. Data were collected from GPs and practice managers in 167 practices. Responses suggested that there was greater availability of services and training for general mental health issues than for suicide prevention specifically. The three key themes which emerged from GP interviews were: barriers accessing primary or secondary mental health services; obstacles faced when referring a patient to mental health services; managing change within mental health care services

**Conclusions:**

Health professionals have an important role to play in preventing suicide. However, GPs expressed concerns about the quality of primary care mental health service provision and difficulties with access to secondary mental health services. Addressing these issues could facilitate future suicide prevention in primary care.

## Background

Suicide is a leading cause of death in England and Wales, accounting for approximately 5000 deaths annually [1,2]. Approximately one-quarter of individuals who complete suicide have been in contact with mental health services [3]. While suicide prevention is clearly important within mental health services, it is not exclusively the remit of any one service [1]. There is good evidence to suggest that initiatives within primary care may contribute to suicide prevention [4-8]. Further, appropriate training for GPs in the identification and treatment of mental health problems has been shown to be effective [9] as has training in suicide prevention [10] (although this has not been found consistently) [11]. As such, it is important to have appropriate services within primary care to effectively manage patients with suicidal behaviour and to ensure access to specialist mental health services when required.

To date, studies on suicide prevention in primary care have focused on the identification, management and assessment of risk [10,12,13] and treatment of depression [14,15]. However, comparatively little is known about what suicide prevention services are available in primary care, or general practitioners' (GPs) experiences of accessing and using these services.

The aims of the current study were to describe services available in general practices for the management of suicidal patients, and to examine GPs views on these services.

## Methods

### Sample

The data collected for this study form part of a larger investigation into health service contacts for a sub-sample of patient suicide cases occurring in the North West of England, collected as part of the National Confidential Inquiry into Suicide and Homicide by People with Mental Illness (Inquiry) [3,4]. The Inquiry collects data on all suicide deaths for individuals who had been in contact with mental health services in the 12 months prior to death [3].

During the study period (2003-2005) we used the Inquiry database to identify 286 general practitioners (GPs) who had registered patients who had died by suicide. As the study was based on Inquiry data all GPs had been the primary care physician for a patient who had also been in contact with mental health services prior to their death. GP details were obtained from local NHS Trusts or from the coroner files relating to the decedent.

We carried out a questionnaire and interview study collecting data on GPs views of suicide prevention generally as well as local mental health service provision. The semi-structured interviews were carried out with GPs consent. The interview schedules were adapted from tools used in previous studies conducted within the Inquiry [16-18]. The interviews ranged between 20 and 40 minutes in duration and took place in GP practices. Practice managers completed the service related questions if GPs were not available (in 5% cases). With the agreement of the participant, interviews were recorded and transcribed verbatim by a member of the research team.

### Data Analysis

#### Descriptive statistics

Quantitative data analyses were conducted using SPSS 15.0 for Windows (SPSS Inc. 2006) [19]. Descriptive statistics are presented including percentages and 95% confidence intervals. When percentages are quoted, these refer to 'valid cases', i.e. those for whom the relevant information was available. Therefore, if an item of information was not known about a person, they were excluded from the analysis of that item. As a result the denominator may vary slightly between analyses.

#### Qualitative analysis

Framework analysis was used to analyse GP interview transcripts [20]. In this approach, one piece of data (e.g. one statement, one theme) is taken and compared with all information for similarities or differences. The analysis was principally conducted by the first author, and also by the third and fourth author. Transcripts were examined across the whole data set as well as in the context of each interview, using thematic analysis. The transcripts were read independently and emergent themes and key issues were discussed. The data were interpreted and reanalyzed within the thematic framework to interpret and structure the component statements.

In some cases practice managers and GPs provided data. Where reference is made to respondents, both practice managers and GPs provided the data and where reference is made to GPs views, this represented GP views only.

## Results

Of the 286 GPs who had registered patients who had died by suicide, 159 (56%) agreed to participate and were interviewed. A GP was unavailable for interview in eight cases (e.g. retired, deceased, left practice). In these cases, the practice manager completed the service structure questionnaire. Therefore, interview data on GPs views on suicide prevention was collected for 159 (56%) cases and data on service availability was collected for 167 (58%) cases.

### Descriptive analysis

The responses to the service questionnaire are shown in Table 1. The majority of practices reported having a psychiatric liaison process. Respondents reported that specific staff training on suicide and self-harm awareness was provided less frequently than training on mental health issues more generally (31% v. 56%, p < 0.001). There were significantly fewer services addressing suicidal ideation and self-harm compared to services for mental health problems more generally (16% v. 74%, p < 0.001).

**Table 1:** GP responses to service structure questionnaire

	Yes % (n) n = 167	No % (n) n = 167	N/k % (n) n = 167
Does this practice have a specific psychiatric liaison process?	85.6 (143)	14.4 (24)	0

Are there any additional services/schemes provided at this practice to deal with mental health issues?	73.7 (123)	26.3 (44)	0

Are there any additional services/schemes provided at this practice to deal with suicidal ideas/DSH?	16.2 (27)	83.8 (140)	0

Are there any services/schemes which you think are needed in relation to mental health issues?	85.6 (143)	13.2 (22)	1.2 (2)

Are there any services/schemes which you think are needed in relation to suicidal ideas/DSH?	51.5 (86)	41.9 (70)	6.6 (11)

Does this practice have any written policies/protocols regarding mental health?	37.1 (62)	51.5 (86)	11.4 (19)

Does this practice have any written policies/protocols regarding suicide/DSH?	24.0 (40)	72.4 (121)	3.6 (6)

Do the staff at this practice receive training on mental health issues?	55.7 (93)	44.3 (74)	0

Do the staff at this practice receive training on DSH/suicide awareness?	30.5 (51)	67.7 (113)	1.8 (3)

Do the staff at this practice receive training on risk assessment for suicide?	29.9 (50)	68.3 (114)	1.8 (3)

Do suicides have an effect on you as a GP?	61.0 (102)	21.2 (35)	17.8 (30)

Is there any support for GPs when patients commit suicide?	25.8 (43)	32.1 (54)	42.1 (70)

Approximately two thirds of respondents reported that they were affected by the suicide of a patient. There was little support for staff in the event of a patient suicide. Support was usually received from work colleagues informally; respondents were not aware of any formal support systems at the time of the suicide.

#### Qualitative analysis of GP interview data

Table 2 shows the themes and subthemes relating to GPs views on mental health service provision with selected key quotes. The findings are discussed more fully below.

**Table 2:** Selected key quotes representing the themes and subthemes relating to GPs views on mental health service provision

Theme	Subtheme	Statement/meaning unit
**Barriers**	Lack of access to Secondary MH services	"Main problem is lack of staff, psychologists, CPNs and now have half the number of psychiatrists in their area than there should be."
		"Cannot refer directly, need to go via CMHT who may send referral back."
	Long waiting lists	"Have a two-tier service for brief intervention such as CBT but waiting times are about 18 months."
		"Long waiting lists for counsellors so GPs do not bother referring."
		"Waiting lists for mild to moderate mental health problems need to be improved as currently very poor."
	Closed lists	"Psychology service was closed for 2 years, no access to psychology in this area."
		"Lack of counsellors and psychologists, 2 year waiting lists and no CBT available."
	Not admitted to inpatient unit	"Waiting times and a lack of beds is a problem. Sometimes patients who are referred for assessment cannot be admitted as there are no beds. Sometimes patients have to wait too long."
	Lack of dual diagnosis services	"Main problem in this area is for alcohol issues as these patients are a high risk for suicide yet they are hard to admit if they need to detoxify."
**Referrals**	Access & Rigid criteria	"GPs do not have quick access to support services within mental health services, especially at early stages where they have no immediate access. This may be due to the CMHT not allowing immediate access as they have very rigid criteria. Therefore need faster assessments for vulnerable patients, especially if the GP has assessed them and thinks they are in need of some treatment."
	Do Not Attend/reply - no follow up	"All referrals go to CMHT who then decide who to access and invite for assessment. If no response from patient the CMHT do not follow up. The referral system is not good."
	Referred back to Primary Care	"Service not good if service feels patients do not need to be seen. CMHT seem to refer patients back, find every reason not to see them - this may be due them being under resourced."
	Under resourced	"No immediate access at initial stages and staff should have more specific training. Provision of CMHT service is based on resources not on patient needs."
	Positive systems in place	"Triage system for prompt assessment of mental health issues. If the GP feels there is a problem, can get it assessed quickly by a mental health worker who will refer the patient for specific treatment."
**Managing Change**	GPs feel unsupported	"Feel very unsupported as GPs. Currently trying to improve services for people with anxiety/depression as if not seen as a major illness referrals will not be seen by anyone."
	Lack of staff & high turnover of staff	"Main problem is lack of staff, psychologists, CPNs and now have half the number of psychiatrists in their area than there should be."
	Community Psychiatric Nurse (CPN) on site	"Was better when CPNs were part of the surgery and not separate as now the SMI criteria is not met by some moderate/low depression cases and they are rejected and do not get seen or reviewed."
		"Very good access to CPN service. If psychotic or urgent case can contact psychiatrist directly. CMHT is on site so can ring duty CPN everyday and they'll sort out referral."
	Crisis Team	"Used to have CPN and psychiatrist attached to the surgery with meetings every month which reduced waiting time to two weeks. Now have to go via CMHT which is not as good, would prefer old system but cannot afford or have access to resources."
		"Better services as some people are not seen by crisis team even if GP has recommended they need to be. Sometimes GP has to really force for patients to be seen. Feels there should be assessments in patients' own environment not only in A&E."

##### (i) Barriers to accessing primary or secondary mental health services

Respondents reported a lack of access to mental health services within both primary and secondary care. Some respondents believed this was a result of the introduction of Community Mental Health Teams (CMHTs), a reduction of onsite mental health care services and lack of resources generally. More specifically, respondents spoke of very limited access to services, which they felt did not meet patient need. Further, waiting lists were often several months long or closed due to excessive demand.

GPs were concerned about the lack of treatment options for patients diagnosed with mild to moderate depression or anxiety. These patients rarely met the criteria for a review by CMHTs and were generally referred back to primary care where there were long waiting lists to access primary care mental health services. GPs felt that primary and secondary mental health services were being targeted towards patients with severe and complex problems while the needs of patients with mild to moderate mental health problems were largely unmet.

There were particular issues in relation to accessing services for the management of suicidal ideation and self-harm. Specifically, primary care services were insufficient in the following ways: lack of access to crisis teams; lack of beds available in some in-patient units; dual diagnosis patients not admitted as a result of intoxication at the time of admission.

##### (ii) Obstacles faced when referring a patient to mental health services

In the previous referral system GPs had been able to directly contact a named psychiatrist about a patient; this rarely happened under the new referral system. The new methods of referral were unpopular with GPs as they felt patients who were referred to see a consultant were sometimes assessed by mental health workers and referred back to primary care services without ever having seen a psychiatrist.

GPs also felt unsupported in their decision-making regarding patient referrals and raised the need for more appropriate and faster assessments for vulnerable patients. Specifically, GPs mentioned the following problems:

▪ CMHTs did not see all patients referred by GPs if they felt the patient did not meet their criteria to be assessed

▪ GPs felt they sometimes had to force CMHTs to consider patients they felt were high priority

▪ CMHTs did not follow up patients who did not attend their assessment appointments, even though some patients may not have been attending due to their mental illness.

However, GPs also acknowledged the pressure CMHTs were under due to high demand and lack of resources.

##### (iii) Managing change within mental health care services

Respondents were critical of the new patient referral system noting that they now had little access to psychiatrists and there was a constant turnover of psychiatric staff. Most practices now referred patients to community psychiatric nurses (CPNs) or psychiatrists via CMHTs, which they felt was not as efficient as the old referral system. The old system involved having a CPN on site and regular contact with psychiatrists to discuss patients (e.g. patients on the severe mental illness (SMI) register).

For urgent mental health assessments under the new referral system GPs referred to mental health crisis teams who were meant to provide a 'hospital at home' service for people with mental illness [21]. Crisis teams, comprised of CPNs, social workers and support workers, are available 24 hours a day, seven days a week to assess patients in line with mental health legislation and provide support and short-term help. GPs that had a good relationship with their local crisis team were very positive about them. However, GPs were less positive about local crisis teams when they had referred patients who were subsequently not assessed by crisis teams; in these instances GPs had to insist their patient be seen. These GPs felt extremely unsupported by local mental health services and felt they had no other treatment options except prescribing medication. GPs complained of an increase in the fragmentation of services, inadequate continuity of care for patients and poor communication between services.

To address concerns about the lack of access to specialist mental health services some practices had employed graduate mental health workers (GMHW) to work on-site. Graduate mental health workers are an additional, specialist service available within primary care settings to provide treatment for patients with mild to moderate mental health needs [22]. Most GPs seemed positive about this service although some were sceptical as they could not offer an equivalent level of mental health care as CPNs or psychiatrists.

### Summary of main findings

GPs in this study raised concerns about the provision of services and training for mental health problems generally and for the prevention of self-harm and suicidal ideation specifically. Two-thirds of GPs were affected by the suicide of their patient, although only a quarter reported being aware of any support available to them. Perhaps unsurprisingly, GPs who were most positive about secondary care mental health services had easy access and good relations with their local mental health services. In these areas GPs reported good communication and liaison between primary care and mental health professionals. Dissatisfied GPs repeatedly stated that they felt services were better when CPNs and psychiatrists were more accessible. The key themes that emerged from interviews were lack of access to mental health services, problems referring patients to these services and working with the changing remit of mental health services.

### Methodological issues

This was a comparatively large study but the findings must be interpreted in the context of a number of limitations. The GPs recruited to the study were a selected group (individuals who had experienced the suicide of a patient who was under the care of mental health services). Such individuals may have different views from GPs who have never experienced a patient suicide or who have experienced the death of a patient by suicide not under the care of specialist services. Those who responded might also differ in important ways from those who did not respond. A systematic comparison of responders and non-responders was not possible making it difficult to comment on potential biases, however our study contained large absolute numbers (higher than previous studies) and similar key issues were consistently raised by our participants. Another limitation was the fact that the study was carried out in a single region in England so the results may not be applicable to other areas with different populations and clinical services. In addition, data were coded by different members of the research team. However, 114 (68%) were validated by another member of the research team - there was agreement in 112 (98%) cases.

### Implications

Health professionals have an important role to play in suicide prevention [1,3]. In this study, many GPs expressed concern about the quality of primary care mental health service provision and difficulties with access to secondary mental health services [22]. Many of these barriers were not specific to suicide prevention, although addressing them could have a positive impact potentially reducing suicide risk among patients who consult GPs prior to suicide.

Many GPs reported that they had not received formal training in self-harm and suicidal ideation. GPs that had experienced a patient suicide commented on the lack of support. Patient suicides can be devastating for clinicians, arousing feelings of guilt, fear and professional inadequacy [23,24]; formal support systems should be readily available [25].

GPs appeared cautious in some cases about referring patients to mental health specialists due to their perceptions of negative outcomes for these individuals, (e.g. patients not being assessed despite a GP referral) and by a lack of access to treatment options (e.g. psychological services) due to long waiting lists. GPs reported that they had to manage patients with a range of mental health problems including those with serious mental illness, even though Community Mental Health Teams (CMHTs) had been introduced to treat this patient group. Strategies have also been introduced to facilitate the management of patients with mild to moderate mental health problems (e.g. graduate mental health workers (GMHW)) [22]. However, implementation of this role has been problematic (e.g. lack of clarity regarding training, management and their clinical role) [26,27]. Further work should look at the impact of these strategies and GPs decision-making to refer patients to mental health specialists.

## Competing interests

LA is the National Director for Health and Criminal Justice, England. NK is Chair of the Guideline Development Group for the new National Institute for Clinical Excellence (NICE) guidelines into self-harm.

## Authors' contributions

The study was principally designed by KW, PS, NK, LA, JS but all authors had input into aspects of study design. Ethical approval was obtained by DDC and KW. Data collection was carried out by PS, AP, DDC, and CM, supported by AW, NS and KW. Initial data manipulation was carried out by PS, AP, DDC and CM, supervised by DW and LC. Data analysis was carried out by PS, AP, DDC and CM, supervised by DW and LC. Clinical input was provided by NS and NK. The manuscript was prepared by PS, KW, and NS, with supervision from NK, LC, JS and LA. All authors commented on drafts of the paper and contributed to the final version.
